# Unveiling the modulation of *Pseudomonas aeruginosa* virulence and biofilm formation by selective histone deacetylase 6 inhibitors

**DOI:** 10.3389/fmicb.2024.1340585

**Published:** 2024-02-02

**Authors:** Simona Barone, Baptiste Mateu, Luigia Turco, Sveva Pelliccia, Francesca Lembo, Vincenzo Summa, Elisabetta Buommino, Margherita Brindisi

**Affiliations:** ^1^Department of Pharmacy, University of Naples Federico II, Naples, Italy; ^2^Department of Precision Medicine, University of Campania Luigi Vanvitelli, Naples, Italy

**Keywords:** antimicrobial resistance, *Pseudomonas aeruginosa*, bacterial biofilm, HDAC6 inhibitors, cystic fibrosis

## Abstract

Bacterial infections represent a key public health issue due to the occurrence of multidrug-resistant bacteria. Recently, the amount of data supporting the dynamic control of epigenetic pathways by environmental cues has triggered research efforts toward the clarification of their role in microbial infections. Among protein post-translational modifications, reversible acetylation is the most implicated in the feedback to environmental stimuli and in cellular homeostasis. Accordingly, the latest studies identified the histone deacetylase 6 (HDAC6) enzyme as a crucial player in the complex molecular machinery underlying bacterial clearance or killing. A very important milestone for the elucidation of the consequence of HDAC6 activity in bacterial infections is herein described, unveiling for the first time the role of a potent HDAC6 inhibitor in interfering with biofilm formation and modulating virulence factors of *P. aeruginosa*. We demonstrated that compound F2F-2020202 affected the production of some important virulence factors in *P. aeruginosa*, namely pyocyanin and rhamnolipids, clearly impairing its ability to form biofilm. Furthermore, evidence of possible QS involvement is supported by differential regulation of specific genes, namely RhlI, phAz1, and qsrO. The data herein obtained also complement and in part explain our previous results with selective HDAC6 inhibitors able to reduce inflammation and bacterial load in chronic infection models recapitulating the cystic fibrosis (CF) phenotype. This study fosters future in-depth investigation to allow the complete elucidation of the molecular mechanisms underlying HDAC6’s role in bacterial infections.

## Graphical abstract

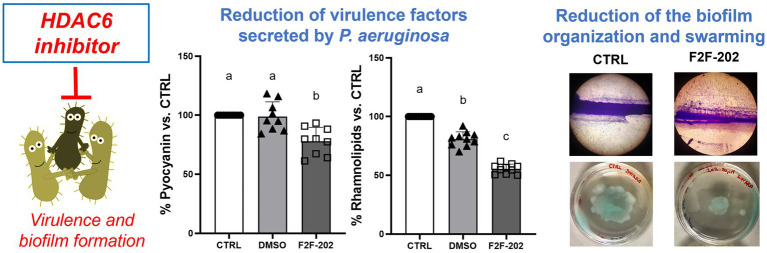

## Introduction

Bacterial infections represent a very serious public health threat due to the emergence of multidrug-resistant bacteria. Common antibiotics are no longer successful against several different microbial species, such as those belonging to the “ESKAPE” pathogens group (Pendleton et al., 2013).

*P. aeruginosa* and *S. aureus*, including methicillin-resistant *S. aureus* [MRSA], are most frequently isolated from the sputum of patients with cystic fibrosis (CF), a genetic disorder commonly involving polymicrobial infections in the respiratory tract with a different distribution in the course of the disease. Predominant bacterial species that colonize CF lung at the early stages of CF include *S. aureus* and *Haemophilus influenzae*, whereas *P. aeruginosa* and the *Burkholderia cepacia* complex are mainly present in the later stages of the disease (Malhotra et al., 2019).

Several virulence factors generated by *P. aeruginosa* play a major role in chronic respiratory infections, and alginate overproduction may drive *P. aeruginosa* coinfection with *S. aureus.* At the same time, both bacteria display a range of competitive and cooperative interactions (Limoli and Hoffman, 2019). Since *P. aeruginosa* and *S. aureus* are common in CF lung, molecules targeting these pathogens or mechanisms associated with their virulence can be relevant in the treatment of polymicrobial infections in pulmonary disease.

Quorum sensing (QS), a cell density-based intercellular communication network, displays key importance in the control of bacterial resistance and virulence, and biofilm formation during bacterial infections. *P. aeruginosa* uses three main quorum sensing (QS) systems, namely Las, Rhl, and Pqs, controlling the production of virulence factors, including proteases and exoenzymes, allowing the bacteria to trigger the infection process in the host tissue (Garcia-Reyes et al., 2020).

Pyocyanin is a key virulence factor generated and profusely secreted by approximately 95% of *P. aeruginosa* isolates, and it has been demonstrated to be essential for full virulence in animal and human airway infection models (Caldwell et al., 2009; Rada and Leto, 2013). Pyocyanin is a phenazine that can cross biological membranes, allowing *P. aeruginosa* to receive and transport electrons arising from the respiration process and to survive in oxygen-limited environments; in the biofilm, pyocyanin creates a redox potential gradient that enhances iron bioavailability, a key factor for biofilm formation. Molecules able to contrast biofilm formation are of great interest in CF disease. Bacteria embedded in the biofilm are safeguarded against immune system responses, contributing to the establishment of a chronic inflammatory status characterized by mucus hypersecretion and abnormal neutrophil recruitment, major factors contributing to pulmonary manifestations of CF patients (Cohen and Prince, 2012). Finally, several pieces of evidence highlight the detrimental effects of pyocyanin on the host immune function, including dodging of defense mechanisms, modulation of multidrug efflux pump expression in *P. aeruginosa* biofilm, and antibiotic resistance (Raju et al., 2018). Thus, the reduction of pyocyanin production may affect the virulence potential of *P. aeruginosa* during airway infections in CF, and pyocyanin could represent a promising target for developing new therapeutic options against *P. aeruginosa* infection.

Recently, the dynamic regulation of epigenetic players by environmental cues emerged as a key factor in bacterial infections and stimulated increasing research efforts toward the clarification of the role of epigenetic players in the infection process (Grabiec and Potempa, 2018). Among protein post-translational modifications, reversible acetylation is the most implicated in the feedback to environmental stimuli and in cellular homeostasis. In this context, the histone deacetylase (HDAC) class of enzymes has been unveiled as key players in the multifaceted molecular mechanisms underlying bacterial clearance or killing.

The QS signal generated by *P. aeruginosa*, namely 2-AA, triggered the reprogramming of immune cells accompanied by increased HDAC1 expression in human THP-1 monocytes. The process was fully reverted by HDAC1 inhibition, thus clearly demonstrating the involvement of this isoform in endorsing tolerance to *P. aeruginosa* (Bandyopadhaya et al., 2016).

As previously stated, metabolites generated by pathogenic bacteria are also able to influence the host acetylation system. Accordingly, it has been shown that short-chain fatty acids (SCFAs) such as propionic and butyric acids secreted by anaerobic bacteria are able to inhibit class I/II HDACs, thus modulating different pathways of immune response (Correa-Oliveira et al., 2016).

Later on, HDAC6 isoform gained increasing attention, since its key role in infection was shown, with particular reference to the immune response associated with the infection process (Hamon and Cossart, 2008; Bierne et al., 2012). HDAC6 differs from other isoforms due to its exclusive cytoplasmic localization (other isoforms mainly reside in the nucleus), which, of course, implies that its substrates are cytoplasmic proteins such as α-tubulin, HSP90, and cortactin. Moreover, in contrast to other HDAC isoforms, HDAC6 features two catalytic domains (Brindisi et al., 2020; Barone et al., 2022).

Accordingly, in 2015, it was demonstrated that only the HDAC6-selective inhibitor TubA and not MS-275, which specifically inhibits class I HDACs, enhanced bacterial killing by macrophages (Ariffin et al., 2015). Similar studies also showed that selective HDAC6 inhibition triggered bacterial clearance, reduced the formation of pro-inflammatory cytokines, reestablished the population of innate immune cells in the bone marrow, and enhanced survival in a sepsis mouse model (Li et al., 2015; Zhao et al., 2016).

This evidence, coupled with the data connecting HDAC6 to the modulation of mitochondrial activity and to the triggering of mitochondrial ROS production, supports a key function for HDAC6 in the regulation of bacterial clearance (Chen et al., 2008; Kamemura et al., 2012; Bai et al., 2015), which strongly differentiates HDAC6 isoform from class I HDACs.

In particular, HDAC6 inhibitors can modulate infection by triggering innate immune-mediated bacterial clearance and/or reducing the damage ascribable to the robust inflammation process associated with the infection. Moreover, these data validate the use of HDAC6-selective inhibitors as an innovative therapeutic option for the treatment of airway chronic inflammation.

To further support this evidence, the outcome of *Hdac6* depletion on both the CF inflammatory response and the bacterial load was recently examined in a model of chronic infection using clinical *P. aeruginosa* to recapitulate the CF phenotype. The loss of *Hdac6* led to increased bacterial clearance in CF mice, thus reestablishing responses to bacterial challenge. Moreover, *Hdac6* depletion limited weight loss and modulated neutrophil recruitment, further validating the benefits of HDAC6-selective inhibitors in diseases featuring chronic airway inflammation, such as CF (Rosenjack et al., 2019). We recently confirmed these data through pharmacological inhibition of HDAC6, demonstrating for the first time the efficacy in reducing bacterial load and inflammatory markers in a mouse model of chronic *P. aeruginosa* infection (Brindisi et al., 2022).

While the role of the so-called acetylome enzymes in bacterial infections is being increasingly explored and clarified, still little information is available on their role in the regulation of virulence and QS processes (Grabiec and Potempa, 2018).

We herein unveil for the first time the role of a very potent and selective HDAC6 inhibitor in the modulation of QS and biofilm formation in *P. aeruginosa.* We demonstrated that F2F-2020202 affected the production of important virulence factors in *P. aeruginosa*, namely pyocyanin and rhamnolipids, clearly impairing its ability to form biofilm. Furthermore, evidence of possible QS involvement is supported by differential regulation of specific genes, namely RhlI, phAz1, and qsrO. The implication of such results is discussed.

## Materials and methods

### Chemicals

F2F-2020202 is a small molecule (chemical formula C_36_H_45_N_5_O_6_, MW = 643 g/mol) designed and synthesized at the Department of Pharmacy, University of Naples Federico II. It displays high potency on HDAC6 (IC_50_ = 4.1 nM) and high isoform selectivity over HDAC1, as a representative of nuclear HDAC isoforms (IC_50_ = 2292.0 nM), with a selectivity index (SI) of 422. The molecule is currently subject to a patent application. Antibiotics were purchased from Sigma-Aldrich (Milan, Italy).

### Strains

*Pseudomonas aeruginosa* ATCC 27853 and *P. aeruginosa* PAO1 (ATCC BAA-47-B1) were obtained from the American Type Culture Collection (Rockville, MD). Each tested compound was dissolved in 100 μL DMSO to give a stock solution (40 mM) and diluted in Mueller-Hinton (MH). Upon sequential dilution, the final DMSO concentration in the assays was well below 2% since literature data demonstrate that 2% DMSO in the assay can modulate QS-associated virulence factors in *P. aeruginosa* (Guo et al., 2016).

### Bacteria antimicrobial susceptibility testing

The minimal inhibitory concentration (MIC) of all the compounds was determined in Mueller–Hinton medium (MH) by the broth microdilution assay, following the procedure already described (Buommino et al., 2021). The compounds were added to bacterial suspension in each well, yielding a final cell concentration of 1 × 10^6^ CFU/mL and a final compound concentration ranging from 3.25 to 100 μM. Negative control wells were set to contain bacteria in Mueller–Hinton broth plus the amount of vehicle (DMSO) used to dilute each compound. Positive controls included 2 μg/mL of tobramycin (TOB 4.27 μM). All antibiotic concentrations reported are according to breakpoint values reported in the EUCAST v.12.0 (The European Committee on Antimicrobial Susceptibility Testing, 2022). The MIC was defined as the lowest concentration of the drug that caused a total inhibition of microbial growth after 24 h incubation time at 37°C. Medium turbidity was measured by a microtiter plate reader (Thermo Scientific Multiskan GO, Waltham, MA, United States) at 595 nm. All the tests were conducted at least three times using independent cell suspensions.

### Motility inhibition assays

Swimming and swarming motilities were performed following a published procedure (Zhou et al., 2018). Briefly, 2 μL of overnight *P. aeruginosa* PAO1 cultures (OD 620 = 0.5) treated with 30 μM compound F2F-2020202 was inoculated at the center of the swimming agar (1% tryptone, 0.5% NaCl, 0.3% agar, pH 7.2) and swarming agar medium (1% tryptone, 0.5% NaCl, 0.5% glucose, 0.5% agar, pH 7.2), respectively. DMSO (0.15%) was used as a negative control. Plates were incubated at 37°C overnight, and migration was then evaluated.

### Biofilm inhibition assay

#### Crystal violet assay

The assay was performed following two procedures, both employing the final crystal violet (CV) staining. First, the assay was conducted in glass test tubes (13×100 mm) as described by Yamamoto et al., with some modifications. Briefly, compound F2F-2020202 was added at a final concentration of 30 μM, at which no growth inhibition was observed, to a volume of 3 mL of medium containing 10^6^ CFU/mL of *P. aeruginosa* PAO1 strain. Control cells were grown in medium broth alone. Negative controls were set to contain bacteria in Mueller–Hinton broth (MH) plus the amount of vehicle (DMSO) used to dilute the compound. After culturing for 24 h at 37°C, the supernatant was gently removed, and the tubes were rinsed with 3 mL of PBS. The biofilm biomass was then measured by staining with 3 mL of 0.1% crystal violet, ensuring that the formed ring pellicle was covered. After 30 min of incubation, the crystal violet was removed, and the biofilm biomass was quantified by adding ethanol (EtOH) 100%. The absorbance was measured at 620 nm using a microtiter plate reader (Thermo Scientific Multiskan GO, Waltham, MA, United States). The percentage of growth inhibition was determined using the following formula: % of biofilm inhibition = ((Control OD – [compound F2F-2020202 or DMSO OD])/Control OD) × 100. [Control represents MH with bacterial inoculum alone]).

*P. aeruginosa* tends to form a characteristic air–liquid interface biofilm. The second protocol was performed to allow the microscopic visualization of *P. aeruginosa* biofilm on glass surfaces (Kasthuri et al., 2022). Briefly, wells containing 1 mL of MH broth with 15 μM of F2F-2020202 or DMSO were used as treated and control groups, respectively. Then, 10^6^ CFU/mL of *P. aeruginosa* PAO1 strain was added to each well. Successively, a 1 cm^2^ glass piece was put in the well diagonal to the broth surface and incubated at 37°C for 24 h. After incubation, the slides were carefully taken with forceps, gently washed three times in sterile distilled water, and then stained as above reported. The unabsorbed dyes were removed by washing the slides in sterile distilled water. After air drying at room temperature, the biofilm architecture of control and treated samples was observed at × 400 magnification (Iris Digital System, Twin Helix).

#### Pyocyanin and rhamnolipid assay

Pyocyanin appears in different colors in media depending on the pH and oxidation status of the culture (Rada and Leto, 2013). To ensure that the 691/600 nm ratio of the untreated bacterial cells was not higher than that of the treated cultures due to a difference in the pH or oxidation status, pyocyanin was first quantified according to Kumar et al. (2014) with some modifications. *P. aeruginosa* PAO1 cultures prepared as above reported (MIC assay) were treated with 30 μM compound F2F-2020202 for 24 h at 37°C. The microbial culture was separately collected from each growth medium and centrifuged at 10,000 rpm for 15 min. The cell-free culture supernatant (CFCS) was extracted with chloroform (5/3, v/v). The organic phase was mixed with 1 mL of hydrochloric acid (0.2 M). After 10 min centrifugation at 4°C, the organic phase was collected. The intensity of the solution color was quantified by measuring its absorbance at the wavelength of 520 nm.

The CFCS was also used for another pyocyanin quantification protocol. The CFCSs of the control and treated groups were collected by centrifugation at 10,000 rpm for 10 min and filtered through a 0.2 μm membrane filter. Pyocyanin concentration was then determined by measuring the absorbance at 691 nm.

For the rhamnolipid assay, *P. aeruginosa strain* PAO1-treated culture was centrifuged (10,000 g for 10 min), the supernatant was collected and acidified to pH 2 (with HCl), and the absorbance was measured at 570 nm.

#### RNA isolation and real-time PCR

*P. aeruginosa* was treated with 30 μM of F2F-2020202 for 24 h. Total RNA was isolated using the GenUp Total RNA kit (biotechrabbit, Berlin, Germany) according to the manufacturer’s instructions. DNA contamination from the total RNA was removed by incubation with DNase I (RNase-free DNase Set, Qiagen, Hilden, Germany). Measuring the A260/A280 nm ratio assessed the nucleic acid purity. To generate cDNA, total RNA was reverse transcribed using RevertUP II Reverse Transcriptase (biotechrabbit, Berlin, Germany) into cDNA using random hexamer primers (Random hexamer, Roche Diagnostics, Monza, Italy) at 48°C for 60 min according to the manufacturer’s instructions. The real-time PCR was carried out using 1 μL of cDNA (5 ng/μl). Expression levels of several quorum sensing genes (see Table 1) were analyzed by quantitative real-time polymerase chain reaction (qRT-PCR) using the CFX96 system (Bio-Rad, Hercules, CA, United States) with the SYBR Green Master Mix kit (Applied Biosystems, Waltham, MA, United States). The thermal cycling parameters were the following: 50°C for 2 min for UDG activation, followed by the activation of the DNA polymerase at 95°C for 2 min, 40 cycles comprising 15 s at 95°C for denaturation, and 1 min at the primer-specific annealing temperature (see Table 1) for annealing and extension. The experiments were carried out in duplicate for each data point, and the ribosomal gene *rpsL* was used as an internal control to normalize all data.

**Table 1:** Primer sequences used for the qRT-PCR and annealing conditions.

Gene	Sequence (5′-3′)		Annealing temperature
LasI	Fw	GGCTGGGACGTTAGTGTCAT	60°C
	Rev	AAAACCTGGGCTTCAGGAGT	
LasR	Fw	CTGTGGATGCTCAAGGACTAC	55°C
	Rev	TCGTAGTCCTGGCTGTCCTT	
RhlI	Fw	AAGGACGTCTTCGCCTACCT	60°C
	Rev	GCAGGCTGGACCAGAATATC	
RhlR	Fw	CATCCGATGCTGATGTCCAACC	55°C
	Rev	ATGATGGCGATTTCCCCGGAAC	
pqsE	Fw	GGATGCCGAATTGGTTTG	53°C
	Rev	GGTCGTAGTGCTTGTGGG	
phzA1	Fw	AACGGTCAGCGGTACAGGGAAAC	60°C
	Rev	ACGAACAGGCTGTGCCGCTGTAAC	
phzA2	Fw	CTGTAACCGTTCGGCCCCCTTCATG	60°C
	Rev	ATGCGAGAGTACCAACGGTTGAAAG	
qsrO	Fw	ATGCTTACGTTTTGGGCTAT	53°C
	Rev	ATGGAAATGGATTCTTTTGAGTT	
rpsL	Fw	GCAACTATCAACCAGCTGGTG	60°C
	Rev	GCTGTGCTCTTGCAGGTTGTG	

### Statistical analysis

Each antimicrobial assay was repeated at least three times. All results of antimicrobial activity are expressed as mean ± standard deviation (S.D.). The results were analyzed using one-way analysis of variance (ANOVA) followed by Tukey’s *post-hoc* comparison tests to verify differences between compounds and concentrations (*p* < 0.05).

## Results and discussion

**Figure 1:**
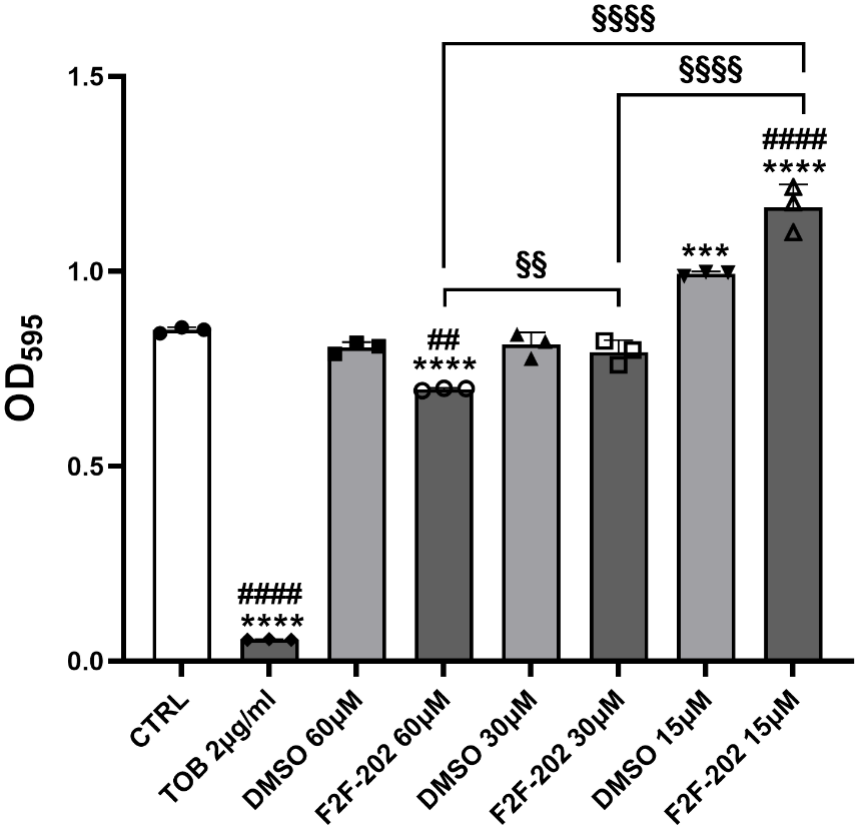
MIC determination of compound F2F-2020202 against *P. aeruginosa* PAO-1. The bacterial growth was determined at different concentrations of compound F2F-2020202 (from here onward reported in figures as F2F-202, 60, 30, and 15 μM) after 24 h incubation time at 37°C. CTRL represents the untreated bacteria. DMSO 60 μM and 30 μM (0.3 and 0.15%, respectively) served as the negative controls. TOB represents tobramycin at 2 μg/mL (4.27 μM). Three replicates were performed. The error bars indicate the mean ± standard deviation of the mean. Statistical differences were determined using ANOVA followed by Tukey’s test (***p < 0.001 and ****p < 0.0001 vs. CTRL; ##*p* < 0.01; ####*p* < 0.0001 vs. DMSO; §*p* < 0.01 and §§§§*p* < 0.0001 for significant differences among the compound F2F-2020202 at different concentrations). Tobramycin showed a significant difference with DMSO at every concentration (####*p* < 0.0001).

Compound F2F-2020202 was first tested for its ability to affect bacterial cell growth on the *P. aeruginosa* PAO-1 strain. As reported in Figure 1, the compound did not induce an evident and significant reduction of cell growth at 30 and 15 μM. Only at 60 μM, a slight reduction of cell growth was observed (growth reduction percentage = 18%).

**Figure 2:**
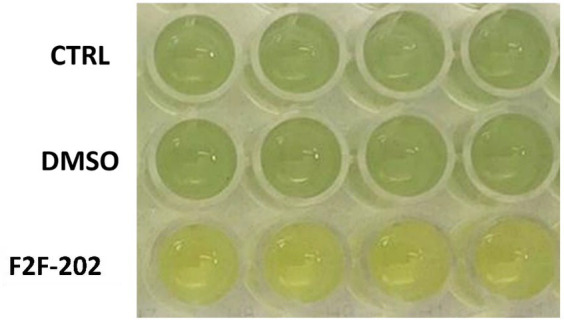
Pyocyanin reduction induced by compound F2F-2020202 at 30 μM. CTRL represents the untreated bacteria. DMSO 30 μM (0.15%) served as the negative control. F2F-202 is the abbreviation for F2F-2020202. The yellow staining of 30 μM F2F-2020202-treated cell media reflects a reduction of pyocyanin production when compared to the control and DMSO-treated cells (green staining). The image is representative of nine different experiments.

However, the 30 μM F2F-2020202-treated cells showed a visible reduction in pyocyanin production (yellow staining of the medium) when compared to the control cells (green staining) (Figure 2). The same result was not observed at the 15 μM (data not shown). Consequently, the impact of compound F2F-2020202 on the production of some PAO-1 virulence factors such as pyocyanin and rhamnolipids was further analyzed using biochemical assays.

**Figure 3:**
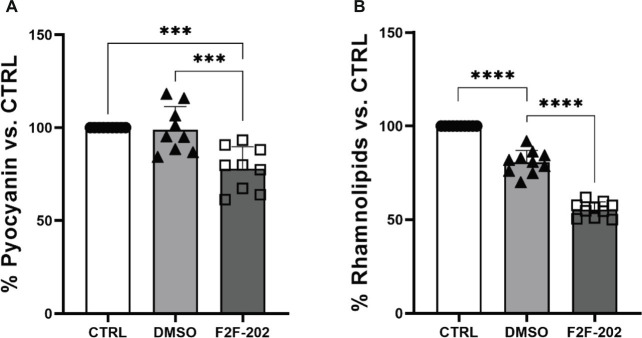
Effect of compound F2F-2020202 on virulence factors secreted by *P. aeruginosa* PAO1. CTRL represents the untreated bacteria. DMSO 30 μM (0.15%) served as the negative control. F2F-202 is the abbreviation for F2F-2020202. The levels of pyocyanin **(A)** and rhamnolipids **(B)** at 30 μM concentration were evaluated. Bar errors showed the standard deviations of nine, nine, and ten replicates, respectively. Statistical differences were determined using ANOVA followed by Tukey’s test (***p < 0.001 and ****p < 0.0001).

Biochemical quantification results confirmed the diminished level of pyocyanin (22% decrease) (Figure 3A) and rhamnolipids (44% decrease) (Figure 3B) in 30 μM treated *P. aeruginosa*. DMSO (0.15%), used as a vehicle, did not affect by itself the production of virulence factors.

**Figure 4:**
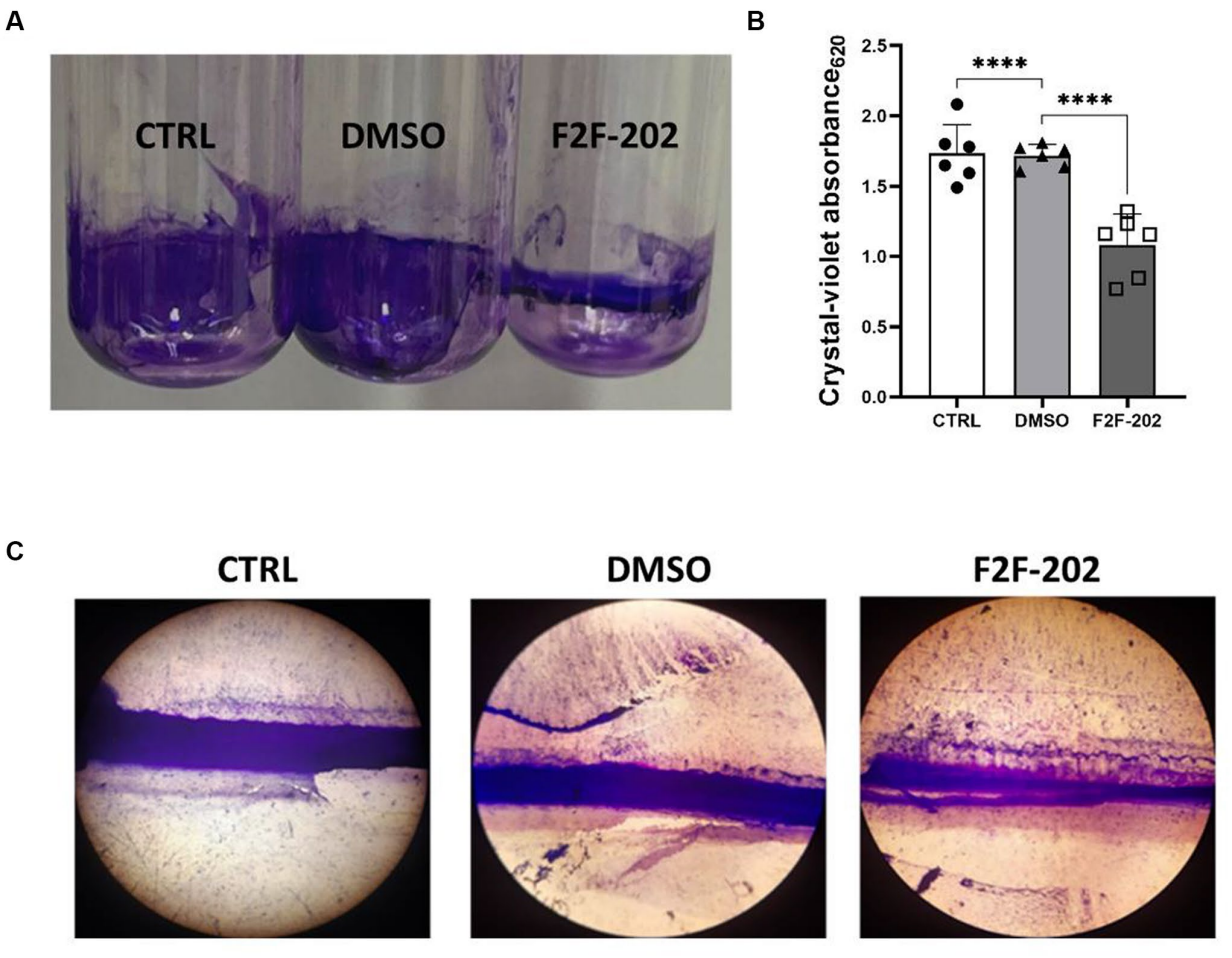
Effect of compound F2F-2020202 at 30 μM on biofilm formation. CTRL represents the untreated bacteria. DMSO 30 μM (0.15%) served as the negative control. F2F-202 is the abbreviation for F2F-2020202. **(A)** The biofilm assay in glass tubes at air–liquid interfaces showed a visible reduction of the biomass attached to the glass walls. **(B)** Crystal violet staining. The absorbance values obtained at 620 nm are presented as mean ± standard deviation. Statistical differences were determined using ANOVA followed by Tukey’s test (****p < 0.0001). **(C)** Representative image of three independent experiments performed on *P. aeruginosa* PAO1 showing the reduction of the biofilm organization and compactness. Images were micrographed using the light microscope.

Pyocyanin is an important *P. aeruginosa* virulence factor capable of directly inducing pulmonary pathophysiology, associated with a decline in lung function, and is essential for the release of extracellular DNA (eDNA), aiding in the formation of biofilm (Das et al., 2013). Additionally, pyocyanin contributes to the dominant colonization of *P. aeruginosa* in the CF lung (Carlsson et al., 2011). Considering the important role of pyocyanin in biofilm formation, we analyzed the potential effect of F2F-2020202 on biofilm inhibition at 30 μM. The treatment with F2F-2020202 significantly affects *P. aeruginosa* biofilm formation, as the pellicle in the glass tube was thin and slightly colored (Figure 4A). A crystal violet assay allowed the estimation of biofilm formation by quantifying the crystal violet uptake by the biofilm matrix. As shown in Figure 4B, treatment with F2F-2020202 reduced biofilm formation by 38% compared to untreated and DMSO-treated cells. We used also a second method to allow the microscopic visualization of *P. aeruginosa* biofilm on glass surfaces. In Figure 4C, biofilm was allowed to form on a 1cm^2^ glass piece. It is evident that F2F-2020202 reduced the thickness of the biofilm, resulting in less organization and compactness compared to DMSO-treated and control cells.

**Figure 5:**
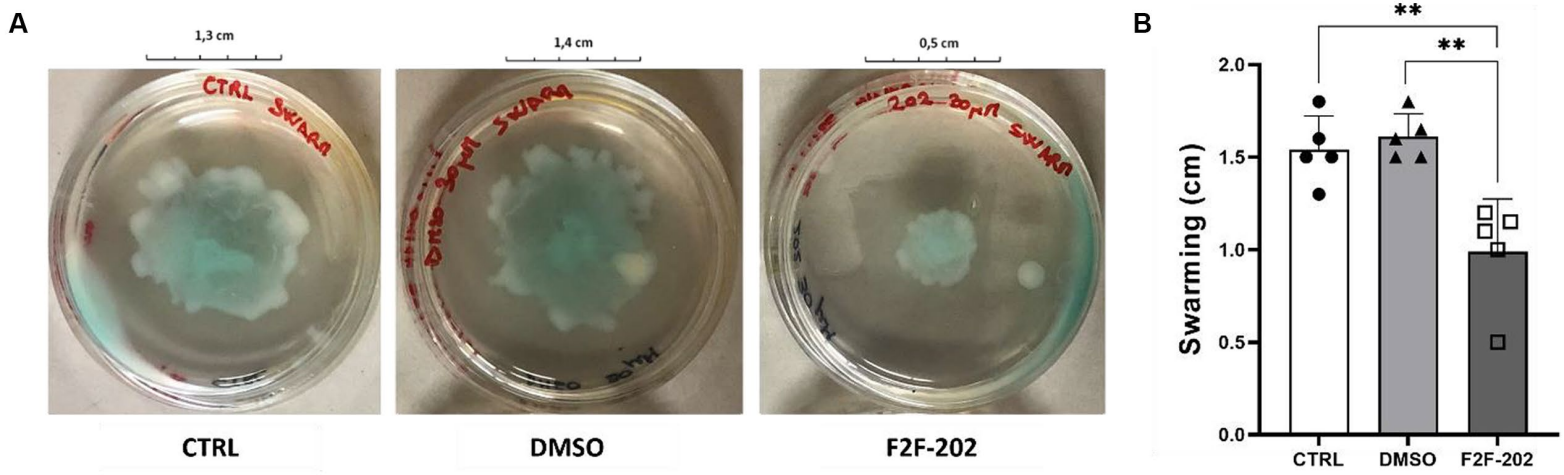
**(A)** The pictures are representative of three independent experiments conducted on *P. aeruginosa* PAO1. CTRL represents the untreated bacteria. DMSO 30 μM (0.15%) served as the negative control. F2F-202 is the abbreviation for F2F-2020202. Compound F2F-2020202 at 30 μM showed a visible reduction in the swarming mobility on plate agar. **(B)** The bar chart shows the effect of compound F2F-2020202 at 30 μM on the swarming motility. Five replicates were done for the statistical comparisons. The measurements are presented as centimeters ± standard deviation. Statistical differences were determined using ANOVA followed by Tukey’s test (**p < 0.01).

Finally, since motility is a key factor in biofilm development, we analyzed the activity of compound F2F-2020202 on swarming motility, an organized form of surface translocation useful in the early stages of biofilm development (Shrout et al., 2006). Swarming motility was affected by treatments with F2F-2020202 at 30 μM, as shown in Figures 5A,B. This result is in accord with the observed reduced rhamnolipid production, a rhamnose-containing glycolipid surface-active molecule with hemolytic activity, encoded by the QS-controlled Rhl genes. The importance of such results relies on the role played by rhamnolipids in chronic and acute *P. aeruginosa* lung infection. They contribute to atelectasis, inhibit the mucociliary transport and ciliary function of the human respiratory system, are implicated in swarming motility, and finally play a crucial role in biofilm formation (Davey et al., 2003).

**Figure 6:**
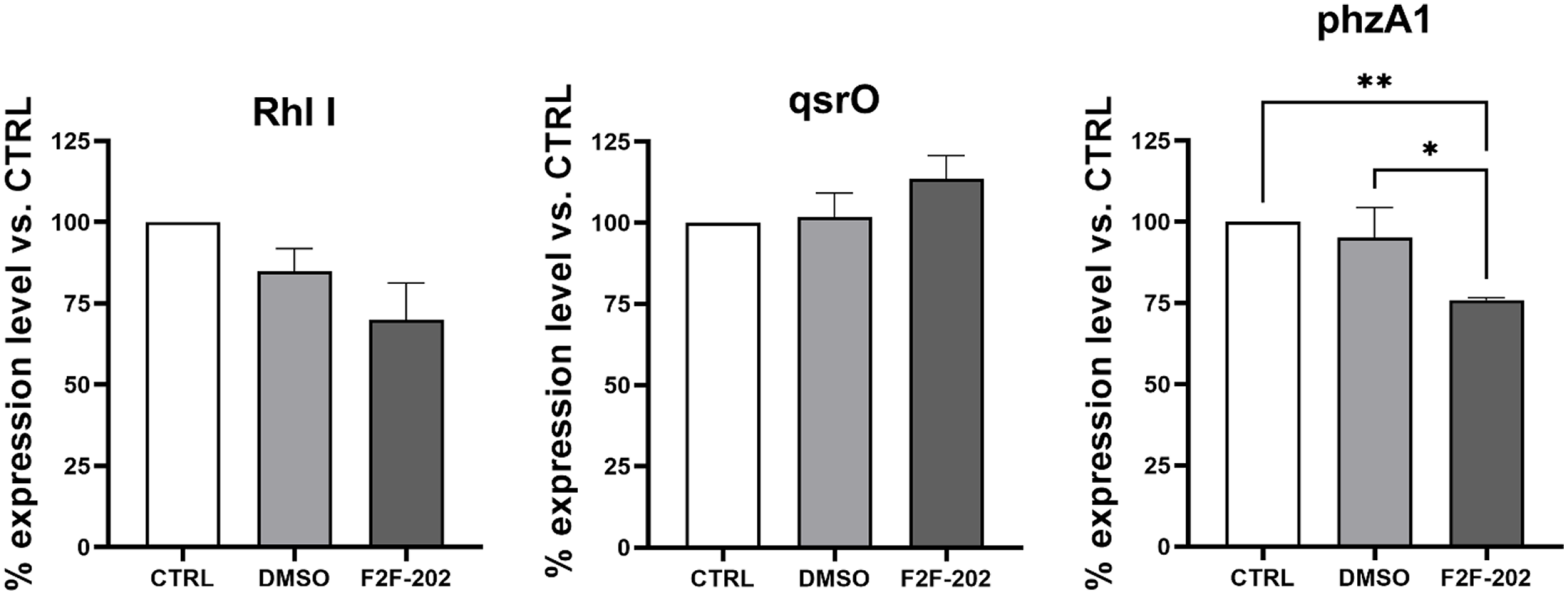
Expression levels of rhlI, qsrO, and phzA1 expressed as a percentage of the expression level of these genes in the control group. CTRL represents the untreated bacteria. F2F-202 is the abbreviation for F2F-2020202. DMSO 30 μM (0.15%) served as the negative control. Three replicates were done for the statistical comparisons. Statistical differences were determined using ANOVA followed by Tukey’s test (*p < 0.05, **p < 0.01).

To confirm the biochemical results herein reported, we sought to determine using real-time PCR the efficiency of F2F-2020202 to modulate some QS-related genes that are involved in the regulation of the above-mentioned virulence factors. We focused our analysis on Las, Rhl, and Pqs. In summary, LasI and RhlI control the synthesis of their autoinducers (AIs) N-(3-oxododecanoyl)-L-homoserine lactone (3-oxo-C12-HSL) and N-butanoyl-L-homoserine lactone (C4-HSL), respectively, which bind to LasR and RhlR. The chemical signaling pathways are hierarchically organized, with Rhl modulated by Las. The third QS network in *P. aeruginosa* is the *Pseudomonas* quinolone signal (Pqs), which is a non-acylated homoserine lactone (AHL)-mediated QS signaling network employing alkyl-4-quinolones (AQs), among which 2-heptyl-3-hydroxy-1H-quinolin-4-one (PQS) and 2-heptyl-1H-quinolin-4-one (HHQ) as signal chemical effectors. These diversified AIs used by the QS network are strongly interconnected and mutually modulate their activities. Upon binding to LasR, RhlR, or PqsR, these signal molecules activate the expressions of QS-related genes of *P. aeruginosa*, controlling the production of virulence factors, including proteases and exoenzymes, allowing the bacteria to trigger the infection process in the host tissue (Garcia-Reyes et al., 2020). QS controls the pyocyanin biosynthesis through a cascade of complex steps orchestrated by two enzymes encoded on two homologous operons phzABCDEFG, named phzA1 and phzA2, whose expression can be repressed by the *qsrO* gene product (Mavrodi et al., 2001; Kohler et al., 2014). The regulator gene *qsrO* plays an important role in the QS system since it can downregulate all QS system regulatory and target genes. Finally, AHLs and HHQs also participate in the modulation of inflammation and immune responses in the host (Caldwell et al., 2009). In this context, molecules targeting QS have been suggested to have a beneficial effect by reducing *P. aeruginosa* virulence factors and, thus, its pathogenicity (Rada and Leto, 2013) and boosting the susceptibility to antibiotics of the bacteria embedded in biofilm (Cohen and Prince, 2012). We analyzed the expression levels of genes coding signal molecule catalyzing enzymes (*pqsE, lasI,* and *rhlI*), the expression levels of genes coding for transcriptional activators binding with signal molecules (*lasR* and *rhlR*), and *qsrO* gene expression, which are the main QS genes investigated in *P. aeruginosa*. Surprisingly, despite the relevant reduction of virulence factors described above, we did not observe a strong modulation of the gene expression (Figure 6). Gene expression levels decreased by 30% for rhlI and 24% for phzA1, while qsrO was induced by 18%. On the contrary, lasI, lasR, rhlR, pqsE, and phzA2 were not modulated (data not shown). With these data in hand, we have tried to provide a rational explanation of the experimental results and to link the QS gene expression to the observed biochemical results. Of course, the biochemical results offer an unambiguous picture of the F2F-2020202 effect on the modulation at the molecular level of QS. Accordingly, upon our preliminary investigation of gene expression, the registered reduction in *rhlI* gene expression might lead to reduced C4-HSL production, inducing an absence of regulation of the hierarchically connected QS genes. This could explain the reduction of pyocyanin and rhamnolipid synthesis, both under the control of *rhlI*. Rhamnolipid regulation is also under the control of *pqsE*, which is involved in bacterial response to PQS and pqsR. Unfortunately, we did not observe a *pqsE* gene expression modulation, thus making even more complex the interpretation of the phenotypic results. Worthy of note, the null mutation in PQS leads to reduced biofilm formation and decreased pyocyanin, elastase, PA-IL lectin, and rhamnolipid production (Yan and Wu, 2019).

However, due to the complexity of the QS system in *P. aeruginosa*, from the results obtained in this study, it is difficult to link the QS gene expression to the observed biochemical results. The partial reduction of some QS gene expressions, as well as the lack of modulation of others, suggests that the observed effects are not directly under the control of the *rhl* system but, rather, linked to a more complex interaction among QS regulators.

## Conclusion

In this study, we showed for the first time the activity of new selective HDAC6 inhibitors in the attenuation of *P. aeruginosa* virulence. Compound F2F-2020202 reduced the production of pyocyanin and rhamnolipids and affected the ability of *P. aeruginosa* to form biofilm. Our results are strongly relevant since they demonstrate for the first time the effect of such inhibitors on the modulation of virulence factors of *P. aeruginosa*.

The data herein obtained can also in part explain our previous results with selective HDAC6 inhibitors showing the reduced inflammation and bacterial load in chronic infection models recapitulating CF phenotype. The significant reduction in pyocyanin and rhamnolipid production, key players in the pathophysiology of CF, and subsequent biofilm reduction should lead to reduced inflammation and recovered protective immune response. This study fosters future in-depth investigation to allow the complete elucidation of the molecular mechanisms underlying F2F-2020202 activity. In particular, targeted RNA sequencing to profile-specific QS transcripts will be helpful in studying and unveiling genes and pathways regulated by HDAC6 inhibitors.

## Data availability statement

The original contributions presented in the study are included in the article/supplementary material, further inquiries can be directed to the corresponding authors.

## Author contributions

SB: Investigation, Writing – review & editing, Formal analysis, Data curation. BM: Formal analysis, Investigation, Writing – review & editing. LT: Investigation, Writing – review & editing. SP: Investigation, Methodology, Writing – review & editing. FL: Formal Analysis, Supervision, Writing – review & editing. VS: Resources, Validation, Writing – review & editing. EB: Conceptualization, Supervision, Writing – original draft. MB: Investigation, Writing – review & editing, Conceptualization, Funding acquisition, Supervision, Writing – original draft.
